# The Chinese Herbal Prescription JieZe-1 Inhibits Membrane Fusion and the Toll-like Receptor Signaling Pathway in a Genital Herpes Mouse Model

**DOI:** 10.3389/fphar.2021.707695

**Published:** 2021-09-24

**Authors:** Qianni Duan, Tong Liu, Cong Huang, Qingqing Shao, Yonggui Ma, Wenjia Wang, Tianli Liu, Jun Sun, Jianguo Fang, Guangying Huang, Zhuo Chen

**Affiliations:** ^1^ Department of TCM, Institute of Integrated Traditional Chinese and Western Medicine, Tongji Hospital, Tongji Medical College, Huazhong University of Science and Technology, Wuhan, China; ^2^ Department of Pharmacy, Tongji Hospital, Tongji Medical College, Huazhong University of Science and Technology, Wuhan, China; ^3^ Department of Biochemistry and Molecular Biology, Tongji Medical College, Huazhong University of Science and Technology, Wuhan, China

**Keywords:** Chinese herbal prescription JieZe-1, genital herpes, herpes simplex virus type 2, membrane fusion, toll-like receptor signaling pathway, immune cells

## Abstract

Chinese herbal prescription JieZe-1 is effective for genital herpes with no visible adverse effects clinically. It showed an excellent anti-HSV-2 effect *in vitro*. However, its mechanism of anti-HSV-2 effect *in vivo* remains unclear. This study was designed to evaluate the anti-HSV-2 effect of JieZe-1 and berberine in a genital herpes mouse model and explore the underlying mechanism*.* The fingerprint of JieZe-1 was determined by high-performance liquid chromatography. First, we optimized a mouse model of genital herpes. Next, the weight, symptom score, morphological changes, viral load, membrane fusion proteins, critical proteins of the Toll-like receptor signaling pathway, cytokines, and immune cells of vaginal tissue in mice at different time points were measured. Finally, we treated the genital herpes mouse model with JieZe-1 gel (2.5, 1.5, and 0.5 g/ml) and tested the above experimental indexes at 12 h and on the 9th day after modeling. JieZe-1 improved the symptoms, weight, and histopathological damage of genital herpes mice, promoted the keratin repair of tissues, and protected organelles to maintain the typical morphology of cells. It downregulated the expression of membrane fusion proteins, critical proteins of the Toll-like receptor signaling pathway, cytokines, and immune cells. The vaginal, vulvar, and spinal cord viral load and vaginal virus shedding were also significantly reduced. In summary, JieZe-1 shows significant anti-HSV-2 efficacy *in vivo*. The mechanism is related to the inhibition of membrane fusion, the Toll-like receptor signaling pathway, inflammatory cytokines, and cellular immunity. However, berberine, the main component of JieZe-1 monarch medicine, showed no efficacy at a concentration of 891.8 μM (0.3 mg/ml).

## Introduction

Herpes simplex virus type 2 (HSV-2) is a linear double-stranded DNA-enveloped virus that mainly infects the skin mucosa of genital, causing genital herpes (GH) (Rubicz et al., 2011). Women of childbearing age are more susceptible to the highest infection rate (James and Kimberlin, 2015). The recognized anti-HSV-2 drugs are acyclovir, penciclovir, cidofovir, and phosphonoformic acid, which mainly work by inhibiting viral DNA replication. No new, significant, and low-toxicity clinical drugs have been identified to treat wild-type and drug-resistant strains (Schleenvoigt et al., 2018). Anti-HSV vaccine research has not yet made a breakthrough (Johnston et al., 2014). HSV-2 has infected approximately 417 million people worldwide, with 19.2 million new cases each year (Looker et al., 2015). The prevention and treatment of HSV have been significant public health issues worldwide.

The adsorption and penetration of HSV-2 into host cells begin with the binding of viral glycoprotein D (gD) to specific receptors (herpesvirus entry mediator ligand protein (HVEM), Nectin-1, Nectin-2, or 3-O-sulfated heparin sulfate) on the cell surface. GB and gH/gL heterodimers are recruited sequentially, resulting in the activation of gH/gL heterodimers. Finally, the fusion activity of gB is upregulated so that it can be directly inserted into the cell membrane through its internal fusion ring (Luo et al., 2015). The recognition of HVEM, Nectin-1, and Nectin-2 by gD triggers membrane fusion (Fu et al., 2018).

The specificity of the Toll-like receptor (TLR) signaling pathway reveals the molecular basis of the body’s recognition of pathogens. After adsorption and penetration, TLR4, which resides on the cell surface, recognizes viral glycoproteins (Lagos et al., 2008). TLR3, 7, and 9, found in the cytoplasm, recognize viral nucleic acids (Paludan et al., 2011; Barber, 2013). The cytoplasmic Toll/interleukin-1 receptor (TIR) domain activates the TLR signaling pathway by interacting with TIR domain adapter myeloid differentiation factor 88 (MyD88)/TIR domain-containing adaptor protein-inducing interferon-β. The TLR signaling pathway includes the MyD88-dependent pathway (most TLRs) and the MyD88-independent pathway (TLR3 and TLR4). Nuclear factor-kappa-B inhibitor alpha (IκBα) is phosphorylated (P-IκBα), triggering its ubiquitination and subsequent degradation. Nuclear factor-kappa-B (NF-κB) is released into the nucleus and binds to cellular DNA to activate the transcription of interleukin-6 (IL-6), tumor necrosis factor-α (TNF-α), interferon-β (IFN-β), and other cytokines to exert early immune response (Dunne and O’Neill, 2003; Hayden and Ghosh, 2004; Yamamoto et al., 2004).

Immune cells are a vital part of the immune response. Natural killer cells (NK cells), dendritic cells (DCs), and other innate immune cells also play essential roles in the first line of defense against HSV-2 infection (Elboim et al., 2013). DCs produce interferon to inhibit virus replication and chemokines from attracting NK cells, which can recognize and lyse HSV-2 infected cells, as well as secrete interferon (Chen et al., 2017). DCs can also present HSV-2 antigens to activate the adaptive immune response of T lymphocytes (Retamal-Díaz A. et al., 2017). CD4^+^ and CD8+T cells, in particular, play primary roles in clearing HSV-2 infection, preventing virus infection in neurons, and reducing latent virus copies.

By drawing lessons from the antiviral research achievements of Traditional Chinese Medicine (TCM), JieZe-1 (JZ-1), an in-hospital preparation of Tongji Hospital (Approval Number: Z20103135), was added and subtracted from Yihuang Decoction, an ancient prescription in “*Fu Qingzhu’s Obstetrics and Gynecology*” during Qing dynasty of China. JZ-1 is composed of 10 herb ingredients (shown in Table 1) and has been widely used for lower genital tract infections with heat-damp symptoms for many years. It shows preventive and therapeutic effects on genital herpes, *Trichomonas* vaginitis, *Candida albicans* vaginitis, and drug-resistant *Ureaplasma urealyticum* infection, with little effect on the dominant vaginal flora (Wei et al., 2008; Chen et al., 2009a, Chen et al., 2009b; Chen et al., 2009c; Chen et al., 2009d; Qiao et al., 2013). JZ-1 also refers to the antiviral research results of TCM. According to the literature reported, *Phellodendron chinense* C.K.Schneid., *Ginkgo biloba* L., *Solanum nigrum* L., *Taraxacum mongolicum* Hand.-Mazz., *Thlaspi arvense* L, *Smilax glabra* Roxb., and *Paeonia* × *suffruticosa* Andrews could resist Zika virus (Robinson et al., 2018), human immunodeficiency virus (Lü et al., 2012), hepatitis C virus (Javed et al., 2011), influenza virus (He et al., 2011), respiratory syncytial virus (Li et al., 2004), HSV-1 (Ooi et al., 2004), and rabies virus (Tu et al., 2018) respectively. All the above scientific evidence gave us reason to believe that the anti-HSV-2 effect of JZ-1 is worthy of being studied. Therefore, our team has done much research on HSV-2. In previous *in vitro* experiments, JZ-1 was found to interfere with HSV-2 adsorption and penetration into host cells by significantly reducing the expression of viral proteins gB, gD, viral protein 16 (VP16), earliest virus-specific infected cell polypeptides 5 (ICP5), and ICP4, improving cell morphology and increasing cell viability (Duan et al., 2020). Furthermore, it also exerts its anti-HSV-2 effect by inducing autophagy via inhibition of the PI3K/Akt/mTOR signaling axis. Moreover, JZ-1 attenuated the increase in inflammatory cytokines that had been induced by HSV-2 infection (Shao et al., 2020). However, its anti-HSV-2 effect and mechanism of action *in vivo* remain unclear.

**TABLE 1:** Composition of Chinese herbal prescription JieZe-1 (JZ-1).

Chinese name	Species name	Family	Plant part	Weight(g)	Ratio (%)	Voucher number
Huangbo	*Phellodendron chinense* C.K.Schneid.	*Rutaceae*	Bark	10	7.1	TJ-1908-318
Baiguo	*Ginkgo biloba* L.	*Ginkgoaceae*	Seed	10	7.1	TJ-1908-112
Longkui	*Solanum nigrum* L.	*Solanaceae*	Fruit, Whole Plant	30	21.4	TJ-1908-154
Pugongying	*Taraxacum mongolicum* Hand.-Mazz.	*Asteraceae*	Whole Plant	15	10.7	TJ-1908-367
Baijiangcao	*Thlaspi arvense *L.	*Brassicaceae*	Aerial Part	30	21.4	TJ-1908-349
Baixianpi	*Dictamnus dasycarpus* Turcz.	*Rutaceae*	Root Bark	10	7.1	TJ-1908-114
Tufuling	*Smilax glabra* Roxb.	*Smilacaceae*	Rhizome	15	10.8	TJ-1908-019
Mudanpi	*Paeonia* × *suffruticosa* Andrews	*Paeoniaceae*	Root Bark	10	7.1	TJ-1908-179
Bohe	*Mentha canadensis* L.	*Lamiaceae*	Aerial Part	10	7.1	TJ-1908-394
bingpian	*Dryobalanops aromatica* C.F.Gaertn.	*Dipterocarpaceae*	Crystal	0.3	0.2	TJ-1908-061

Based on the membrane fusion effect, combined with the regulation of the TLR signaling pathway and immune cells, this paper intends to study the molecular mechanism of JZ-1 interfering with HSV-2 infection in mice, providing scientific insights into JZ-1 against GH.

## Materials and Methods

### Cell Culture and Virus Preparation

The African green monkey kidney cell line (Vero) was purchased from the China Center for Type Culture Collection (CCTCC, Wuhan, China) and cultured in Dulbecco’s modified Eagle’s medium (DMEM) (Thermo Scientific, Waltham, MA, United States) supplemented with 10% fetal bovine serum (FBS) (Invitrogen, Carlsbad, CA, United States). The cells were grown in monolayers, and the medium was changed to the fresh medium every 48 h. HSV-2 strain 333, originally isolated from a primary genital lesion from a patient at the Baylor College of Medicine (Houston, TX, United States), was used in this study because the strain has been widely used in mouse and guinea pig studies and is pathogenic in these species (Lo et al., 2018; López-Muñoz et al., 2018). The HSV-2 strain was purchased from Guangzhou Biotest Biotechnology Development Co., Ltd. (Guangdong, China) and was propagated in Vero cells with DMEM containing 2% FBS. The cells were subjected to three freeze-thaw cycles when more than 80% of them were floating. The supernatants were stored at −80°C after centrifugation. The original HSV-2 suspension was measured by the TCID50 method and plaque assay and diluted to 1 × 10^6^–1 × 10^8^ TCID50/0.1 ml or 2 × 10^4^–2 × 10^6^ PFU/ml before use. Based on the relevant results, 1 × 10^7^ TCID50/0.1 ml (2 × 10^5^ PFU/ml) was chosen as the dose for follow-up experiments.

### Chemicals

A phytopharmacological research should define positive controls and use preferably standard drugs from clinics, which must have been validated and used over the years (Heinrich et al., 2020). As a commonly used clinical anti-HSV-2 drug, acyclovir was used as a positive control drug in the study. The acyclovir (ACV > 99%, Hubei Wushi Pharmaceutical Co., Ltd., Anlu, China) was prepared as 133.2 mM (30 mg/ml) gels before use.

### Preparation of JZ-1 Gel and Berberine Gel

Basing on the principle of scientific nomenclature for plants (Rivera et al., 2014; Heinrich et al., 2020), the full species name including authorities and family, used plant part, and the ratio of the drugs were recorded in Table 1 through consultation with Kew Medicinal Plant Names Services (MPNS, http://mpns.kew.org/mpns-portal/). All the medicinal materials were purchased from Hubei Shengdetang Prepared Slices of Chinese Crudo Drug Co., Ltd. (Xiaogan, China), which complies with the requirements of Chinese Good Manufacturing Practices for Pharmaceutical Products certified by the People’s Republic of China (Supplementary File 2). The quality inspection reports of medicinal materials were provided in Supplementary File 3 (labeled in English). The plant name, batch number, locality, date, inspector’s name, inspection basis, the nature of the samples, and the methodology (using TLC) for determining the identity were reported in the reports. Furthermore, the medicinal materials were also identified by the Associate Professor of Pharmacy Yonggui Ma and Professor of Pharmacy Jianguo Fang. Voucher specimens were prepared for identification and deposited in the traditional medicine collection center for Department Pharmacy of Tongji Hospital (Tongji Medical College, Huazhong University of Science and Technology). The voucher number was presented in Table 1. JZ-1 was prepared as described previously (Duan et al., 2020). Ethanol was selected as an extraction solvent since most components of JZ-1 are alcohol soluble. When the ethanol was recycled, the extract was concentrated to a relative density of 1.20 under 60°C. Carbomer was dissolved with ddH_2_O and added to an appropriate amount of final mixed liquid, and *Dryobalanops aromatica* C.F.Gaertn. (dissolved in anhydrous ethanol) was added. Sodium hydroxide solution (5 M) was added to adjust the pH of the carbomer to form a gel. As a part of “the 10th Five-Year National Science and Technology Project” (Approval Number: 2004BA709B13-02), the procedure selection, the best preparation conditions, and stability of JZ-1 lotion and vaginal gel have been researched by our team in previous studies. JZ-1 gel showed stable efficacy in the stability test performed by our team (Supplementary File 1). Berberine, as the main component of the monarch drug of JZ-1, was also used in our study to determine whether it can exert the same antiviral effect as the Chinese herbal prescription JieZe-1. Berberine (HPLC ≥ 98%, Sigma-Aldrich, St Louis, MO, United States) was prepared as 891.8 μM (0.3 mg/ml) gels before use.

### High-Performance Liquid Chromatography (HPLC) Analysis of JZ-1

For the analysis, the standard products were purchased from Solarbio Science & Technology Co., Ltd. (HPLC ≥ 98%, Beijing, China). D-(-)-quinic acid, trigonelline, and citric acid were prepared at 5 mg/ml with ultrapure water. Berberine, luteolin, caffeic acid, apocynin, taxifolin, and ferulic acid were prepared at 2.5 mg/ml with 50% methanol aqueous solution. Then take these solutions to make a 1,000 µl mix. The resulting solution was filtered through a 0.22 μm membrane filter before HPLC injection. HPLC was performed using an Agilent 1,260 Infinity Ⅱ system (Agilent Technologies, Santa Clara, CA, United States) equipped with a DAD detector and a Welch Ultimate XB C18 (250 mm × 4.6 mm; 5 µm). The mobile phase included 0.2% phosphoric acid plus 0.4% sodium 1-heptanesulfonate (A) and methanol (B) at a flow speed of 1.0 ml/min in the condition of a column temperature of 30°C. The detection wavelength was set at 214 nm. The reference standard sample injection volume was 10 µl. The gradient elution was as follows: 0–5 min (100–100% A, 0%–0% B), 5–60 min (100–60% A, 0%–40% B), 60–120 min (60–30% A, 40–70% B), 120–125 min (30–30% A, 70–70% B), 125–125.1 min (30–100% A, 70–0% B), 125.1–132 min (100–100% A, 0–0% B). The chromatographic data and peak area scores were collected and analyzed using ChemStation software.

### Animals

All the animal experiments were carried out in a class II biosafety laboratory (BSL-2) under the Guidelines for Care and Use of Experimental Animals (Ministry of Science and Technology of China, 2011) and were approved by the Experimental Animal Ethics Committee of Tongji Medical College of Huazhong University of Science and Technology in December 2018 (IACUC Number: 840). BALB/c female mice (9 weeks, 20 ± 2 g) were purchased from Beijing Vital River Laboratory Animal Technology Co., Ltd. (Beijing, China). The mice were fed adaptively for 1 week under a 12 h light/dark cycle at 24°C with free access to water and food. The mice were randomly assigned to 10 in each group.

### Modeling Method

Progesterone increases susceptibility to genital herpes infection (Kaushic et al., 2003). The mice were intramuscularly injected with 150 μl of progesterone for 5 days. On the day of modeling, after anesthesia, the mouse vagina was rubbed repeatedly 50 times and injected with 20 μl of blank gel and then 20 μl of HSV-2 suspension. The symptom score was graded as follows: 0 points, asymptomatic; 1 point, moderate swelling of the vulva; 2 points, small ulcers of the vulva (<5 mm); 3 points, moderate ulcer of the vulva (5–10 mm); 4 points, large ulcer of the vulva (>10 mm); 5 points, death. For symptoms between two levels, ±0.5 points were recorded (Table 2). Samples were taken from the anesthetized mice at 12 h and the 1st, 3rd, 5th, 7th, 9th, 12th, and 14th days after modeling.

**TABLE 2:** Improved symptom score of mice with genital HSV-2 infection.

Symptom score	Corresponding symptoms
0	Asymptomatic (no symptoms such as redness and swelling of vulva, depilation, ulcer, etc.)
0.5	Increased vaginal secretions, slightly swelled vulva, no ulcer
1	Moderate redness and swelling of vulva, no ulcer
1.5	Severe redness and swelling of vulva, perineal protrusion, no ulcer
2	Small ulcers of vulva and surrounding tissue (diameter < 5 mm)
2.5	Small ulcers of vulva and surrounding tissue (diameter < 5 mm) with redness and swelling, hair removal, pus, etc.
3	Moderate ulcer of vulva and surrounding tissue (5 mm ≤ diameter ≤ 10 mm)
3.5	Moderate ulcer of vulva and surrounding tissue (5 mm ≤ diameter ≤ 10 mm) with redness and swelling, hair removal, pus, etc.
4	Large ulcer of vulva and surrounding tissue (10 mm < diameter)
4.5	Large ulcer of vulva and surrounding tissue (10 mm < diameter). Or hind limb paralysis, lower body stiffness, loss of normal movement ability
5	Death (extreme weight loss, cold body, weak breathing, dying or death)

### Drug Administration Method

**FIGURE 1:**
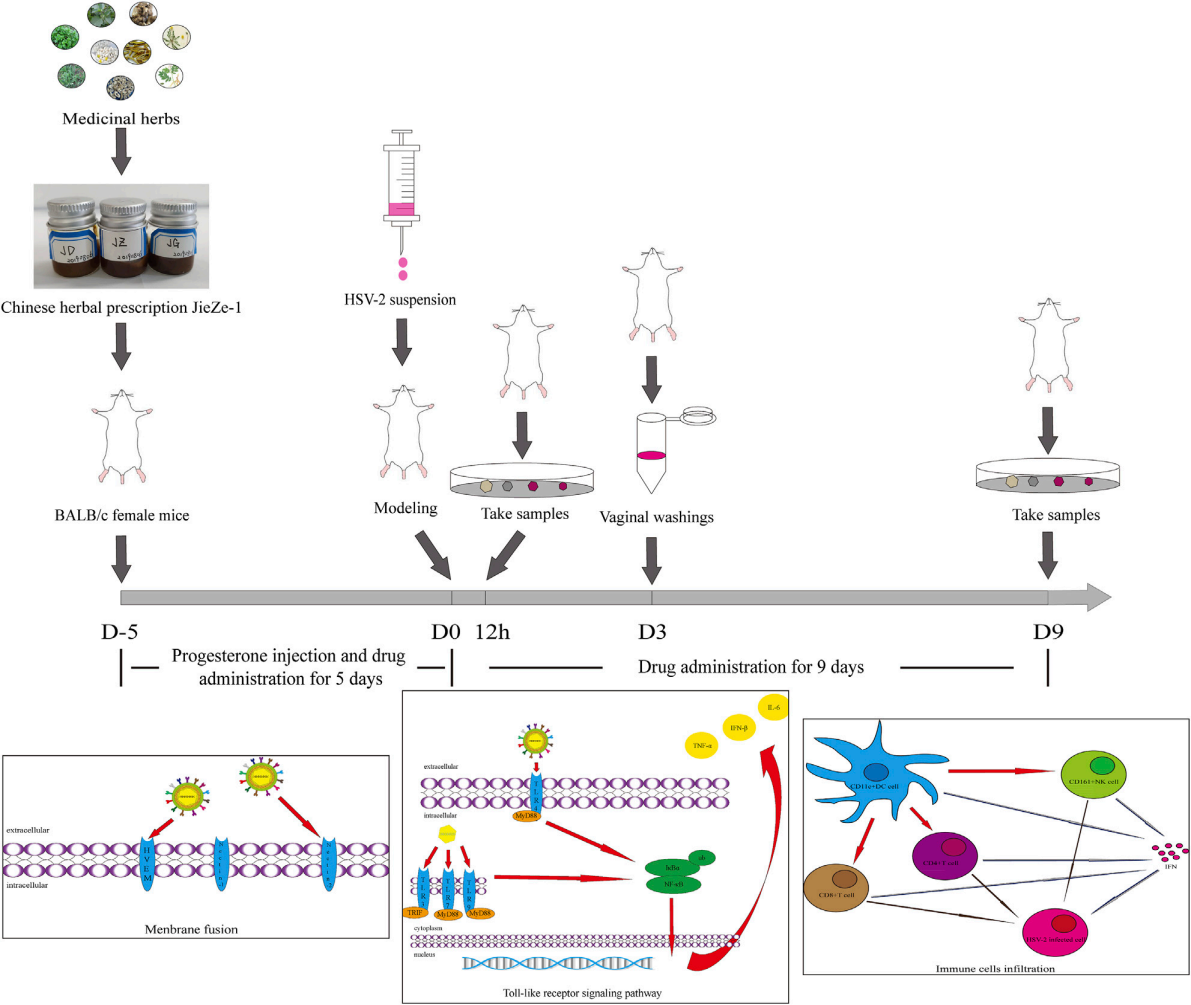
The graphic illustration of the experiment.

We controlled different variables to explore the drug administration method. Different concentrations of JZ-1 (0.5–2.5 g/ml) and administration cycle (9, 12, or 14 days) were used to treat mice with genital herpes. Non-anesthetized mice are active and often squeeze a part of the drug gel out. Anesthesia can significantly increase the residence time of the gel. However, frequent anesthesia can be harmful to mice. We compared the effects of anesthesia and no anesthesia on the efficacy of the drug administration. Based on the relevant results, we selected three concentration gradients, 2.5, 1.5, and 0.5 g/ml, for follow-up experiments. We administered the drug gel without anesthesia to the mice for 14 days, including 5 days before modeling and 9 days after modeling. In addition to progesterone injection, the mice were administered 20 μl of gel (JZ-1 gel, berberine gel, or blank gel) twice a day for 5 days and then were modeled using the previous method. At 12 h, half of the mice in each group were anesthetized and sampled. The other mice were given the gel twice a day in different groups and were continued to be observed. The vaginal washings were collected on day 3. On day 9, we anesthetized and sampled the remaining mice. The experimental protocol was also shown in Figure 1.

### Virus TCID50 Detection

Vero cells were inoculated in a 96-well plate and grown in monolayers. The HSV-2 suspension was diluted 10 times and inoculated with Vero cells. The cytopathic effect (CPE) in each well was observed every day until it no longer increased. The TCID50 was calculated according to the Reed–Muench method (Reed and Muench, 1938).

### Plaque Assays

Vero cells were inoculated in a 24-well plate and grown in monolayers. The HSV-2 suspension (or mouse vaginal washing filtered using a 0.22 μm aseptic filter) was diluted 10 times and inoculated with monolayer Vero cells for 2 h. The supernatant was discarded, and DMEM containing 1% methylcellulose was added to continue the culture until the number of plaques no longer increased. The cells were then fixed with paraformaldehyde and stained with crystal violet solution (Servicebio, Wuhan, China). After washing, plaque formation was observed.

### Western Blotting

The vaginal and vulvar tissues were homogenized in RIPA lysis buffer with protease and phosphatase inhibitors. The concentration of total protein was determined using the BCA kit (Servicebio, Wuhan, China). The loading buffer was added to the extracts, followed by boiling for 10 min. The proteins were separated by 8–12% SDS-PAGE and transferred to a nitrocellulose membrane (Merck Millipore, Billerica, MA, United States). The membrane was sealed in skim milk and then incubated with primary antibodies against gD, ICP5, VP16, Nectin-1, Nectin-2, HVEM, TLR3, TLR9 (Abcam, Cambridge, United Kingdom), TLR4, TLR7 (Absin, Shanghai, China), MyD88 (ABclonal, Wuhan, China), IκBα, and P-IκBα (CST, Danvers, MA, United States) and secondary antibody (LI-COR, Lincoln, NE, United States). Finally, the Odyssey instrument (LI-COR, Lincoln, NE, United States) was used to acquire images on 700 or 800 nm channels.

### Enzyme-Linked Immunosorbent Assay (ELISA)

IL-6, IFN-β, and TNF-α ELISA kits were purchased from Neobioscience Biological Technology Co., Ltd. (Shenzhen, China). The supernatant from the mouse vaginal tissue homogenate was extracted and evaluated using ELISA kits according to the manufacturer’s protocol. In order to normalize the data, we calculated the data along with their own total protein amount to obtain a ratio.

### Histological Morphology and Immunohistochemistry

Vaginal and vulvar paraffin sections were stained with hematoxylin and eosin to assess the pathological changes. Immunohistochemistry was used to detect the infiltration of immune cells. Tissue sections were dewaxed, hydrated, antigen repaired, membrane permeated, and sealed. After incubating with primary antibodies against CD4, CD8, CD11c, and CD161 and secondary antibody (Servicebio, Wuhan, China) successively, the tissue sections were stained, restained, differentiated, dehydrated, cleared, sealed, and photographed for analysis.

### Real-Time Quantitative PCR

Total RNA was extracted from tissues using Trizol reagent (Servicebio, Wuhan, China). The purity and concentration of the total RNA were detected by the NanoDrop 2000 system (Thermo Scientific, Waltham, MA, United States). The cDNA was synthesized by reverse transcribing 2 μg of RNA using the Hifair^®^ Ⅱ 1st Strand cDNA Synthesis Kit (Yeasen Biotech, Shanghai, China) in a Master-cycler gradient PCR apparatus (Eppendorf, Hamburg, Germany). Next, PCR was performed in a 20 μl volume containing 2 μl of cDNA, 8 μl of diluted and mixed primers, and 10 μl of SYBR Green Master Mix (Yeasen Biotech, Shanghai, China) using the LightCycler 96 System (Roche, Basel, Switzerland). The primers used are shown in Table 3 (Namvar et al., 2005), and the relative expression of HSV-2 gB mRNA was calculated using the Log10 (2^−△△Ct^) method.

**TABLE 3:** The sequences of primers for qPCR.

Gene	Primers
HSV-2 gB forward	5’-TGC​AGT​TTA​CGT​ATA​ACC​ACA​TAC​AGC-3’
HSV-2 gB reverse	5’-AGC​TTG​CGG​GCC​TCG​TT-3’
GAPDH forward	5’-CCT​CGT​CCC​GTA​GAC​AAA​ATG-3’
GAPDH reverse	5’-TGA​GGT​CAA​TGA​AGG​GGT​CGT-3’

### Transmission Electron Microscope (TEM)

The vaginal and vulvar tissues of 1 mm^3^ were fixed in 2.5% glutaraldehyde and 1% osmium tetroxide. After dehydrating in ethanol, the tissues were permeated with propylene oxide-epoxy resin compound and embedded with epoxy resin. The sample was then sliced using an ultra-micro slicer. Sample slices (50 nm) were stained with 1% uranyl acetate and lead citrate and then were observed and photographed by TEM (H-7000FA; Hitachi, Tokyo, Japan).

### Statistical Analysis

All the data were presented as means ± standard error (SEM), and SPSS 22.0 software was used for statistical analysis. The data fitting a normal distribution was statistically analyzed using one-way ANOVA. *p < 0.05* was regarded as statistically significant.

## Results

### HPLC Analysis of JZ-1

**FIGURE 2:**
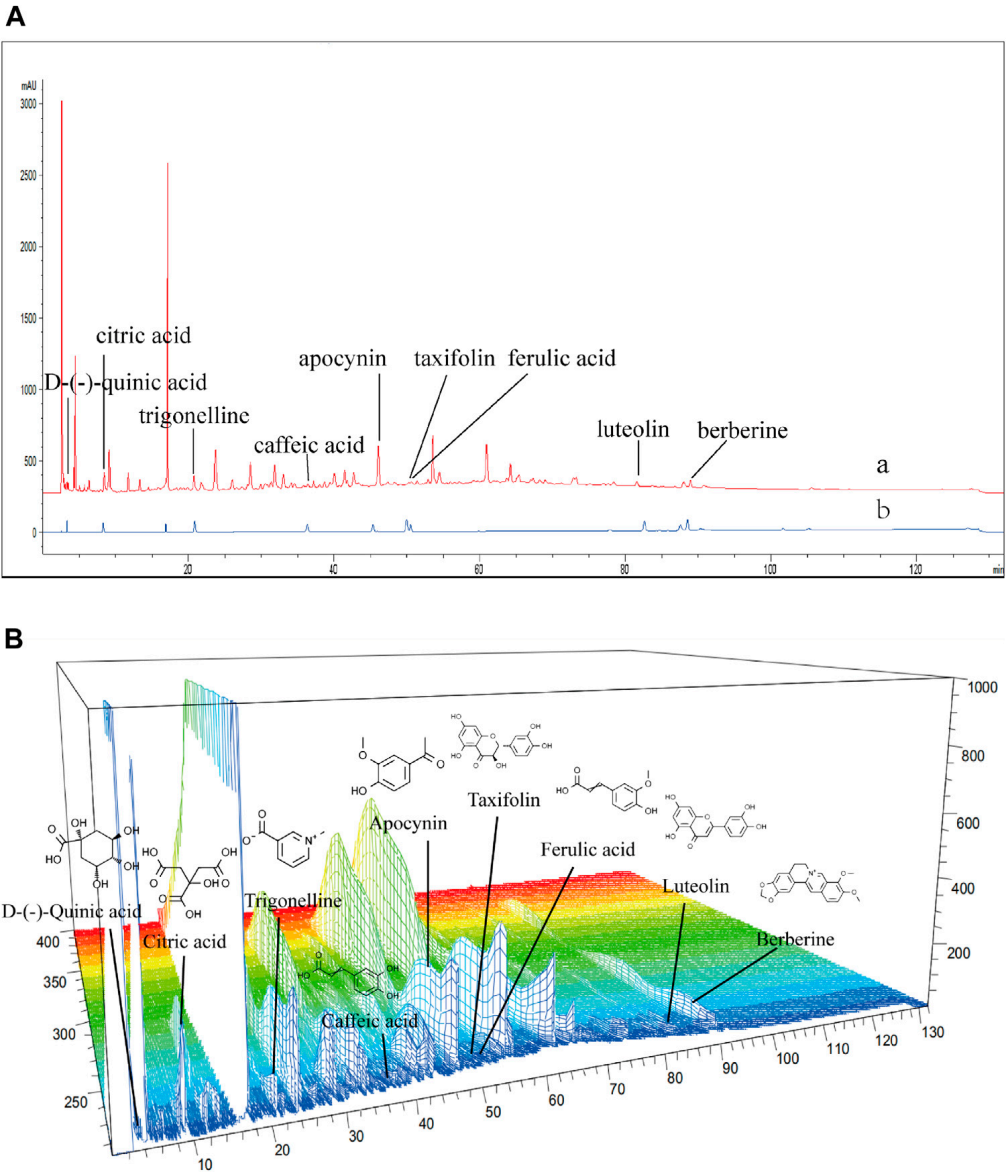
HPLC fingerprinting of JZ-1. **(A)** HPLC fingerprint chromatograms of the JZ-1 (a) and the reference standards (b). **(B)** 3D-HPLC fingerprint chromatograms of the JZ-1.

The high-performance liquid chromatography results of JZ-1 are shown in Figure 2. The nine component chromatographic peaks in JZ-1 were confirmed, comparing the retention times and UV spectrum: D-(-)-quinic acid, citric acid, trigonelline, caffeic acid, apocynin, taxifolin, ferulic acid, luteolin, and berberine. The content of each analyte is shown in Table 4.

**TABLE 4:** Content of nine analytes in JZ-1.

Analyte	Content in mix (μg/ml)	Peak areain mix (mAU*s)	Peak area in JZ-1 (mAU*s)	Content in JZ-1 (μg/ml)	Content in JZ-1 (μM)
D-(-)-Quinic acid	1,250	344.86600	323.89920	1,141.42447	5,939.7
Citric acid	1,250	682.80060	1,608.82605	2,906.93432	15,130.8
Trigonelline	50	985.99890	1,610.65710	78.50905	572.4737
Caffeic acid	25	935.88611	474.04410	12.24522	67.9686
Apocynin	25	764.73834	4,783.61719	147.15459	885.5665
Taxifolin	25	1,388.26794	225.52815	3.95647	13.004
Ferulic acid	25	868.52405	398.51459	11.27769	58.0785
Luteolin	50	1,308.73914	577.26355	21.49175	75.083
Berberine	50	1,104.40137	856.66675	37.97280	112.8799

### Establishment of a Mouse Model of Genital Herpes

**FIGURE 3:**
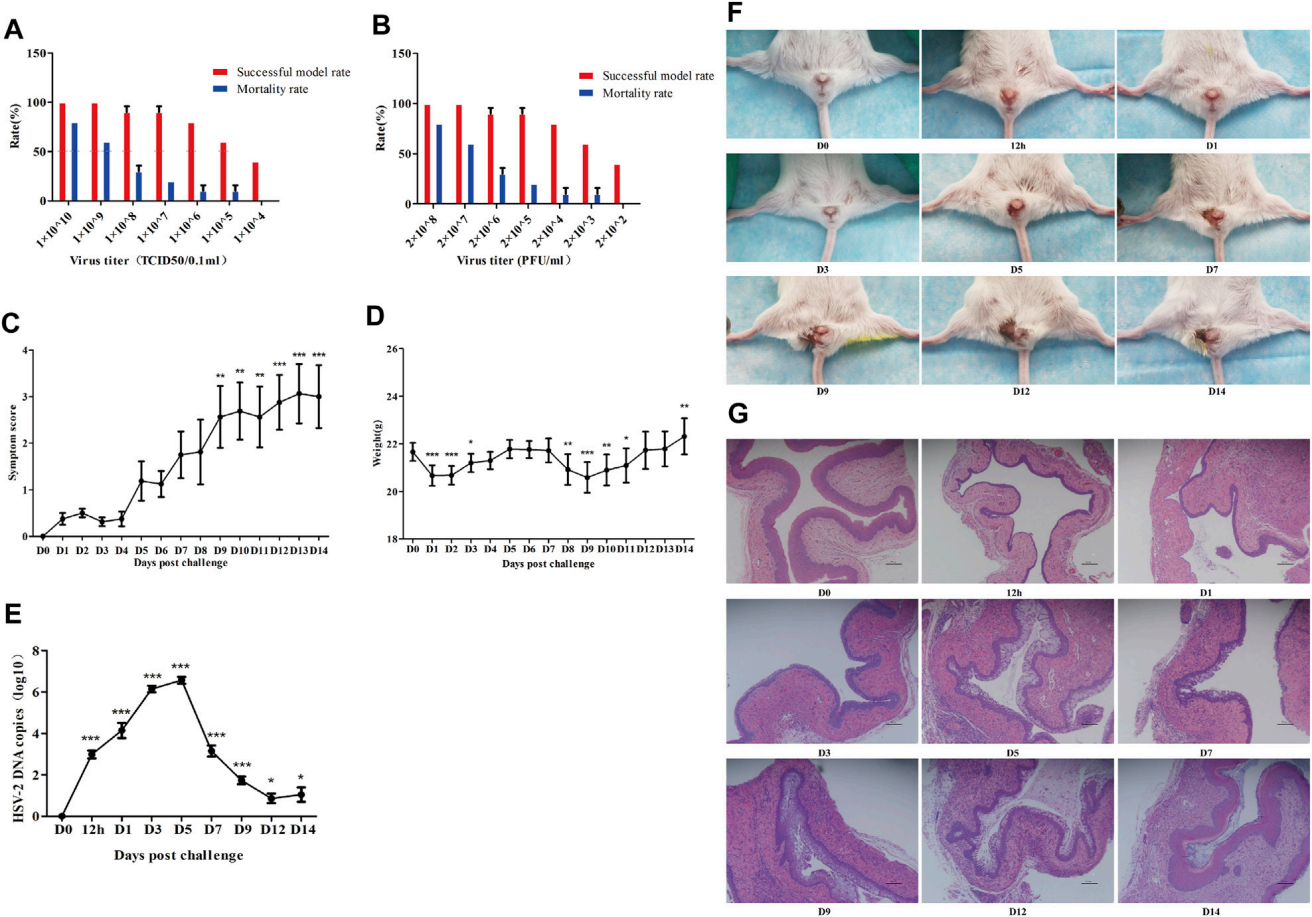
Establishment of a mouse model of genital herpes. The model was established and optimized with multiple batches of mice. Successful model and mortality rates at different virus titers **(A, B)** were observed. Furthermore, symptoms score **(C)**, body weight **(D)**, viral load (HSV-2 gB mRNA) **(E)**, Symptom images **(F)**, vagina histomorphology (×100) **(G)** of mice at different time points after modeling were evaluated. D0: control group. 12 h: the 12 h after modeling. D1: the 1st day after modeling, et al. *p<0.05, **p<0.01, ***p<0.001 vs*.* D0 group.

A high virus titer leads to high mortality in mice, while a low virus titer leads to a low successful modeling rate. When the viral titer was between 1 × 10^6^–1 × 10^8^ TCID50/0.1 ml and 2 × 10^4^–2 × 10^6^ PFU/ml, a stable mouse model of genital herpes could be established with a more than 80% successful modeling rate and a less than 20% death rate (Figures 3A, B). The above results indicate the feasibility and stability of the modeling method of the genital herpes mouse model in this experiment. Then we recorded the changes of indexes aforesaid within 2 weeks after the establishment of the mouse model of genital herpes to gain an in-depth understanding of the disease progress. The symptom (Figures 3C, F) and pathological manifestations (Figure 3G) showed that, after HSV-2 infection, inflammation increased slightly from 12 h to day 1, and then subsided. The vaginal viral load (HSV-2 gB mRNA) increased and reached a peak from day 3 to day 5 (Figure 3E). The symptoms increased rapidly from day 5 and reached a peak on day 9. The vaginal mucosa began to show vacuolar degeneration and necrosis, punctate hyperemia, lymphocyte infiltration, and proliferation of capillaries. From day 9 to day 14, the symptoms were maintained at a high level. The body weight changed with symptoms, and its trend was opposite to that of the symptom score (Figure 3D).

### Changes in the Experimental Indexes Over Time in GH Mice

**FIGURE 4:**
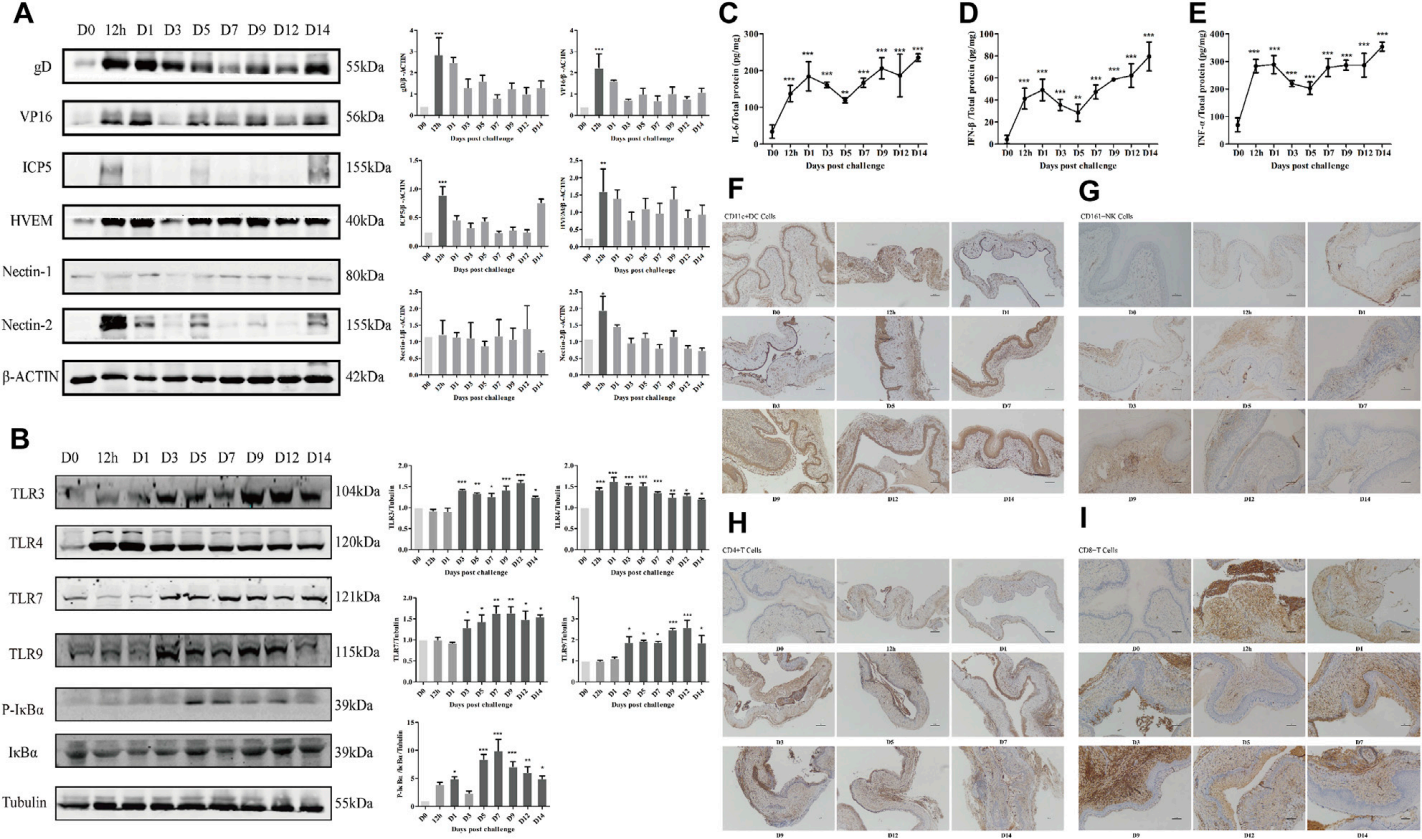
Changes of experimental indexes in vaginal tissue at different time points after modeling in GH mice. The expression of membrane fusion-related proteins **(A)**, TLR signaling pathway-related proteins **(B)**, IL-6 **(C)**, IFN-β **(D)**, TNF-α **(E)**, and the infiltration of CD11c+DC **(F)**, CD161+NK **(G)**, CD4+T **(H)**, CD8+T **(I)** cells over time within 2 weeks after modeling were measured. *p<0.05, **p<0.01, ***p<0.001 *vs.* D0 group.

At 12 h, many virions were injected into the vagina to infect vaginal epithelial cells through abraded mucosa, and the expression of gD, VP16, ICP5, HVEM, and Nectin-2 in vaginal tissue reached a peak and then showed a downward trend (Figure 4A), while the expression of Nectin-1 showed no significant difference over time. Therefore, we chose 12 h after modeling as the time point to explore the anti-HSV-2 membrane fusion effect of JZ-1. After adsorption and penetration, HSV-2 is recognized by TLR on the cell membrane and cytoplasm in vaginal tissue. The expression of TLR4 from 12 h, TLR3, TLR7, and TLR9 from day 3, and P-IκBα/IκBα from day 5 in the model group were significantly higher than those in the control group. TLR4 expression showed a downward trend, and TLR3, TLR7, and TLR9 showed an upward trend (Figure 4B). The expression of IL-6, IFN-β, and TNF-α (Figures 4C–E), as well as the infiltration of DCs, NK cells, and T cells (Figures 4F–I), in the model group, were significantly higher than those in the control group. The first peak was at 12 h to day 1, decreased slightly from day 1 to day 5, and then increased again until day 14. All indicators were highly expressed on day 9, so we chose the 9th day after modeling as the time point to explore the anti-GH effect and mechanism of JZ-1 *in vivo*.

### Exploration of the Effective Concentration and Administration Mode of JZ-1

**FIGURE 5:**
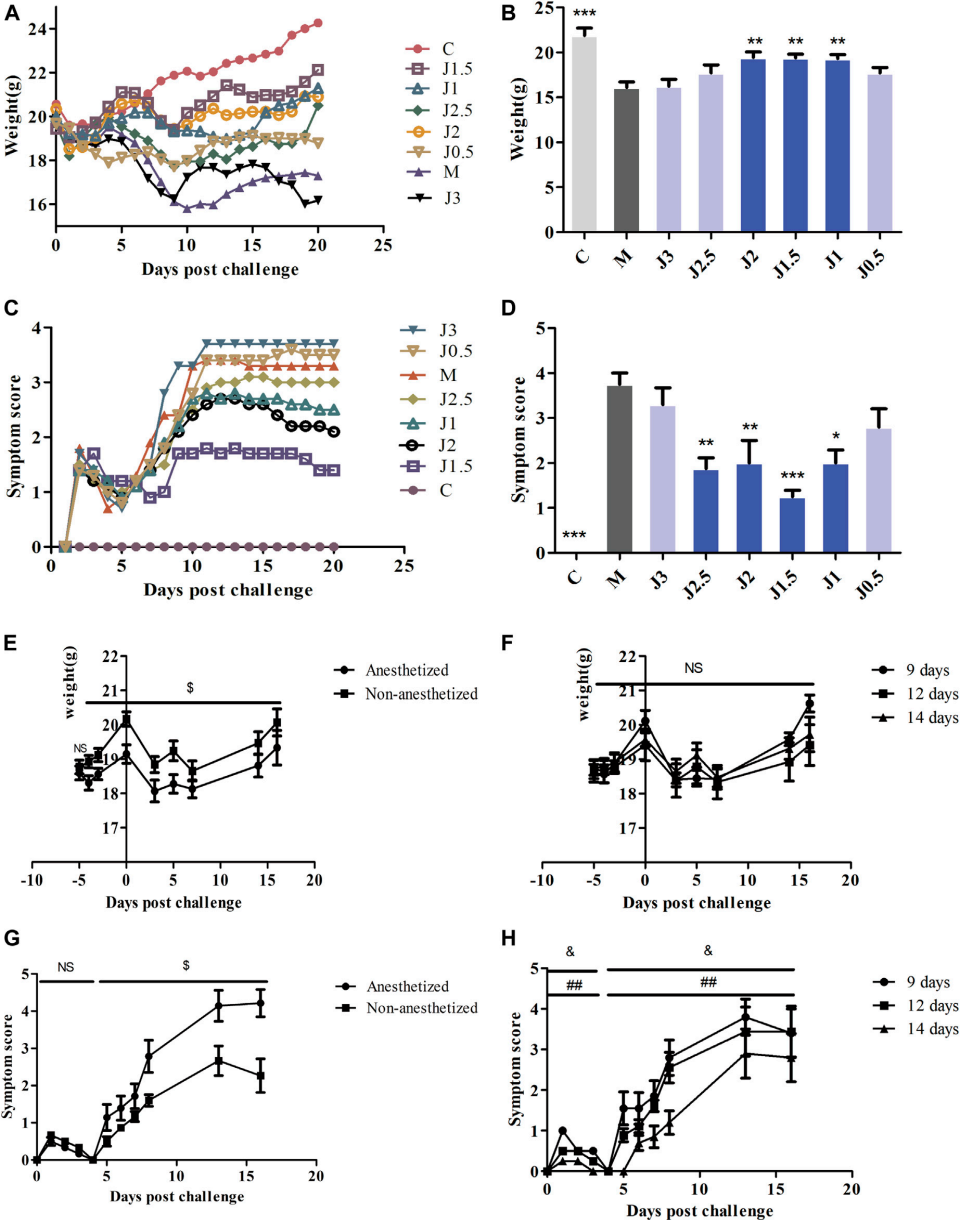
Exploration of the effective concentration and administration mode of JZ-1. The effect of different concentrations JZ-1 on body weight **(A, B)** and symptom score **(C, D)** of GH mice was explored firstly, and the data of **(B, D)** was from day 9. Then, the effect of anesthesia and JZ-1 administration days on body weight **(E, F)** and symptom score **(G, H)** of GH mice was explored. C: control group, M: model group. J3, J2.5, J2, J1.5, J1, J0.5: 3, 2.5, 2, 1.5, 1, 0.5 g/ml JZ-1 group. 14, 12, 9 days: drug administration for 5, 3, 0 days before modeling plus 9 days after modeling. *p<0.05, **p<0.01, ***p<0.001 *vs*. M group, $*p*<0.05 (Anesthetized *vs*. Non-anesthetized), & *p*<0.05 (14 days *vs*. 12 days), ##*p*<0.01 (14 days *vs*. 9 days).

An explanation of the rationale should be provided for the selection of doses, route and frequency of drug administration (Heinrich et al., 2020). We explored the method and concentration of JZ-1 administration. The body weight in the 0.5–2.5 g/ml JZ-1 groups decreased slowly from day 1 to day 12 (Figure 5A,B), and the vulvar symptom score in 1–2.5 g/ml JZ-1 groups were lower than those in the model group (Figures 5C,D). It was found that 2.5, 2, 1.5, 1, and 0.5 g/ml JZ-1 had a certain effect on maintaining the bodyweight of genital herpes mice, and the first four concentrations of JZ-1 could alleviate the vulvar symptoms of genital herpes mice. From day 13 to day 20, the bodyweight of mice in the JZ-1 groups increased significantly, and the symptom score showed a downward trend, suggesting that JZ-1 promotes the healing of genital ulcers. Comparing the trend of body weight and symptom score, the state of mice without anesthesia was better than that in mice with anesthesia (Figures 5E,G). JZ-1 administration for 14 days was better than that for 12 and 9 days (Figures 5F,H). So we selected three concentration gradients, 2.5, 1.5, and 0.5 g/ml, for follow-up experiments. By improving and prolonging the cycle of drug administration, we administered the drugs without anesthesia to the mice for 14 days, including 5 days before modeling and 9 days after modeling.

### Effect of JZ-1 on GH Mice

**FIGURE 6:**
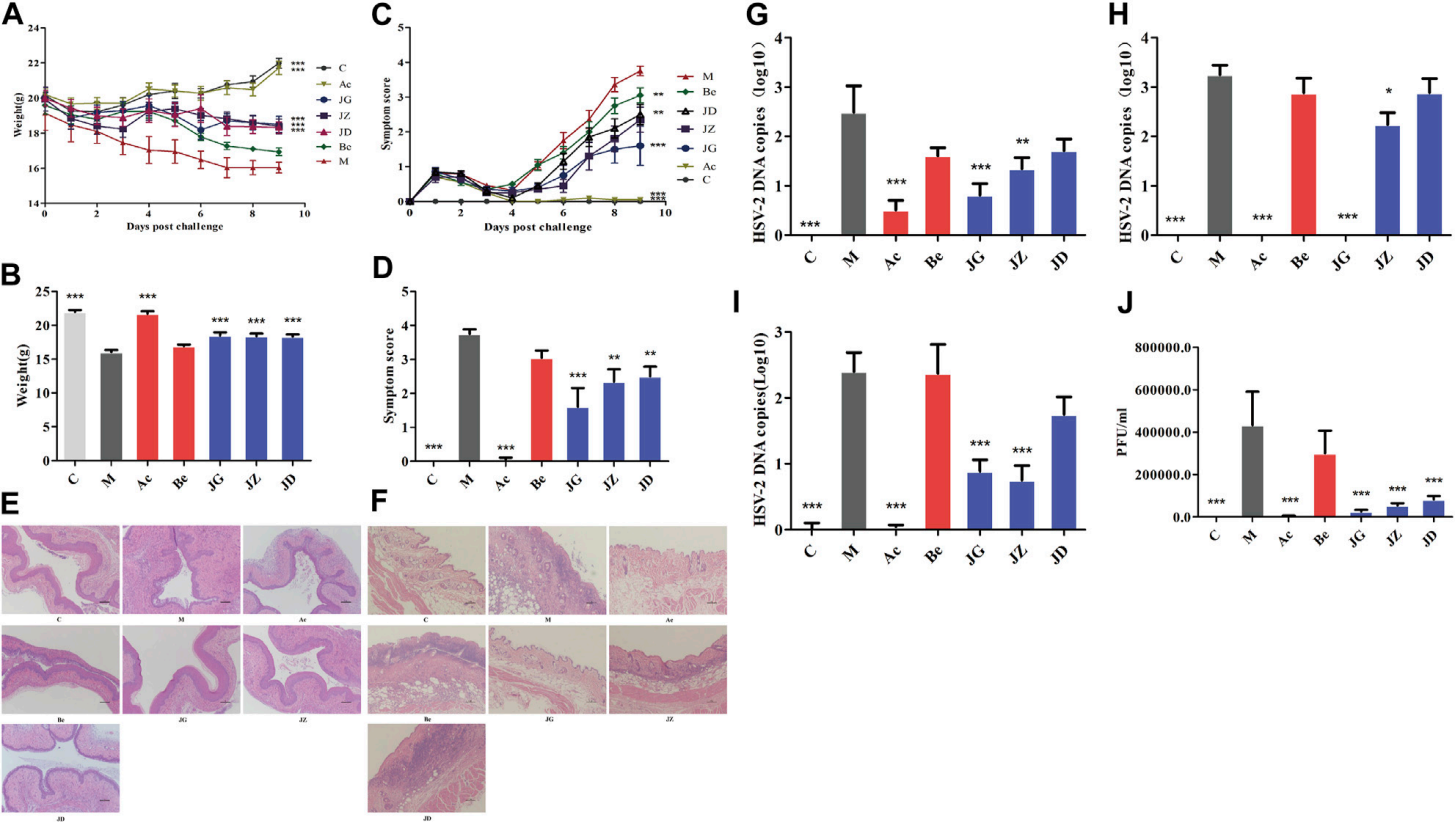
Effect of JZ-1 on GH mice. Effect of JZ-1 on body weight **(A, B)**, symptoms score **(C, D)**, vagina **(E)** and vulva **(F)** histomorphology (×100), vagina **(G)**, vulva **(H)**, and spinal cord **(I)** viral load, virus titer of vaginal washings **(J)** of GH mice was evaluated. The data of **(B, D)** showed the statistical analysis results of body weight and symptoms score on day 9. The data of **(E-I)** was also from day 9, and **(J)** was from day 3. C, control group; M, model group; Ac, acyclovir group; Be, berberine group; JG, JZ, JD: 2.5, 1.5, and 0.5 g/ml JZ-1 group. *p<0.05, **p<0.01, ***p<0.001 *vs.* M group.

In the model group, the vaginal mucosa of mice showed severe pathological manifestations as described previously (Figure 6E), and the vulvar epidermis showed discrete spongiosis, glandular atrophy, multinucleated giant cells, and cytopathic effects on day 9 (Figure 6F). The body weight (Figures 6A,B) of mice decreased. The symptom score (Figures 6C,D), virus titer of vaginal washings (Figure 6J), and the viral load of the vagina (Figure 6G), vulva (Figure 6H), and spinal cord (Figure 6I) increased. Acyclovir and JZ-1 reversed these changes, particularly JZ-1 at 2.5 g/ml. They significantly alleviated vacuolar degeneration and necrosis of the vaginal and vulvar tissues caused by the virus, reduced the infiltration of inflammatory cells, and promoted tissue keratinization and repair.

#### Effect of JZ-1 on ultrastructural changes in the vaginal and vulvar tissues of GH mice

**FIGURE 7:**
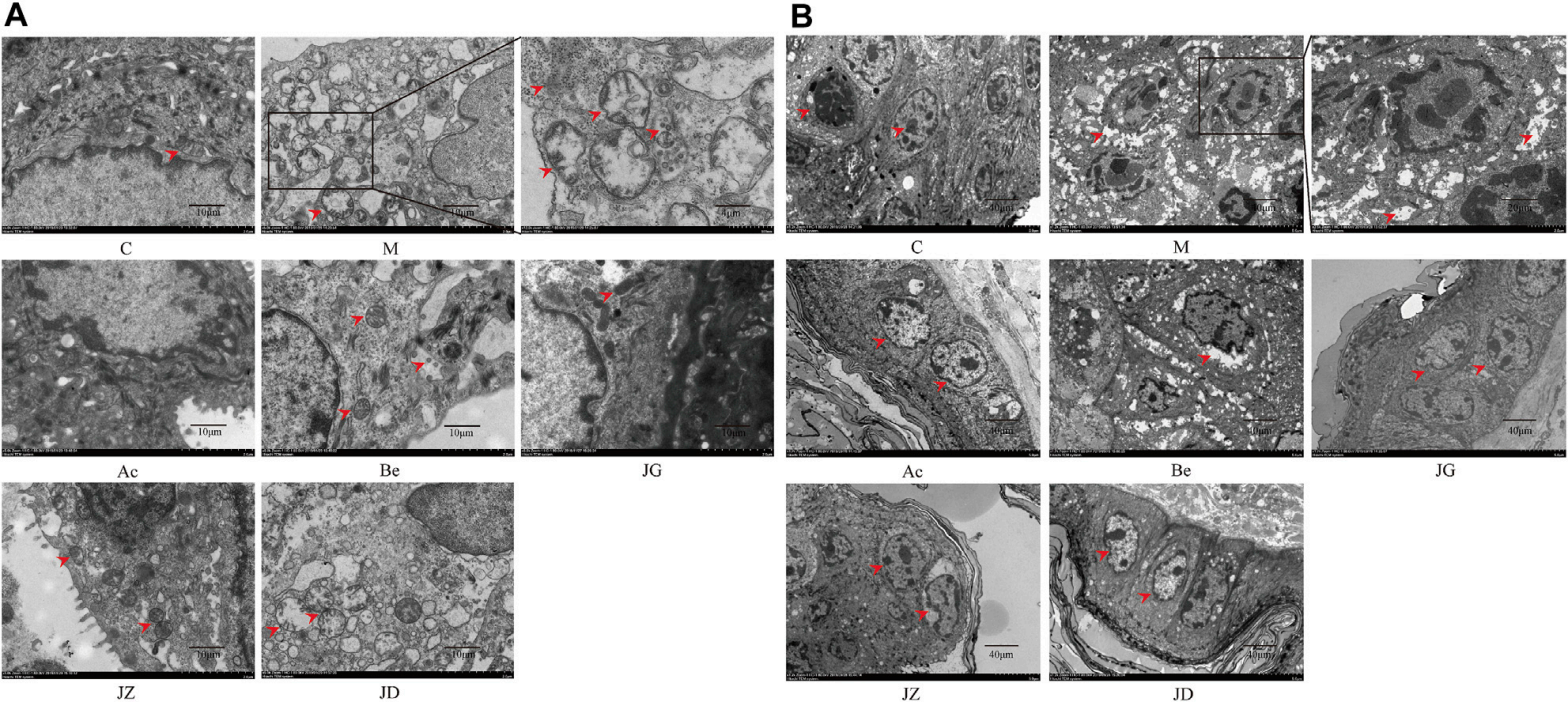
Effect of JZ-1 on ultrapathological changes of vaginal **(A)** and vulvar **(B)** tissues in GH mice. On day 9, after modeling, the characteristic ultrapathological changes of vaginal **(A)** and vulvar **(B)** tissues in GH mice were observed by TEM. JZ-1 alleviated pyknosis and intensely staining of nuclear, reduced the concentration of chromatin, spongiosis changes of cytoplasm, and the number of lysosomes and autophagosomes. The arrows in the diagram showed the pyknosis and intensely staining of nuclear, the concentration of chromatin, spongiosis changes of cytoplasm, the lysosomes, and the autophagosomes. C, control group; M, model group; Ac, acyclovir group; Be, berberine group; JG, JZ, JD: 2.5, 1.5, and 0.5 g/ml JZ-1 group.

For further observation, we detected the effect of JZ-1 on ultrapathological changes of the vagina and vulvar tissues using TEM. As shown in Figure 7, on day 9, the perinuclear spaces of vaginal and vulvar cells in the model group were highly dilated and showed discrete spongiosis changes under TEM. The nucleus was pyknotic and deeply stained, and the chromatin was concentrated. Rough endoplasmic reticulum expansion, swelling, and degranulation could also be seen. The Golgi apparatus swelled, and the mitochondria showed high swelling, rupture, vacuolation, and lipofuscin increase. Many free ribosomes can be seen, and the lysosomes and autophagosomes increased compensatively. Many monolayer vesicles appeared in the cells, and virions were observed in the vesicles. The characteristic ultrastructural changes in the acyclovir group and JZ-1 groups were significantly fewer than those in the model group, and the acyclovir group and JZ-1 2.5 g/ml group were closest to the control group.

#### Effect of JZ-1 on the expression of membrane fusion-related proteins in vaginal and vulvar tissues of GH mice

**FIGURE 8:**
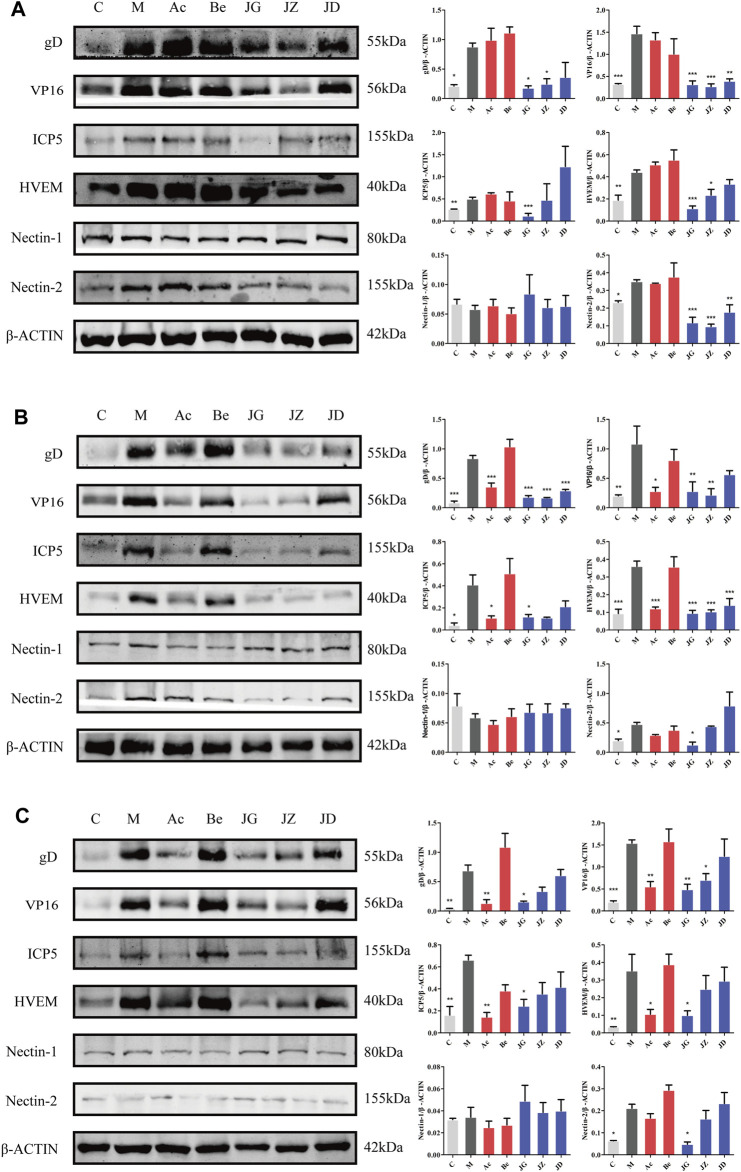
Effect of JZ-1 on the expression of membrane fusion-related proteins in vaginal **(A, B)** and vulvar **(C)** tissues of GH mice. On 12 h **(A)** and day 9 **(B, C)** after modeling, the key proteins of membrane fusion, including gD, VP16, ICP5, HVEM, and Nectin-2, were measured by western blot. JZ-1 inhibited virus adsorption and penetration by downregulating the expression of membrane fusion-related proteins. C, control group; M, model group; Ac, acyclovir group; Be, berberine group; JG, JZ, JD: 2.5, 1.5, and 0.5 g/ml JZ-1 group. *p<0.05, **p<0.01, ***p<0.001 *vs.* M group.

First of all, we cut in from the membrane fusion in the early stage of infection. The expressions of gD, VP16, ICP5, HVEM, and Nectin-2 were increased in the model group and decreased significantly in the JZ-1 groups. At 12 h, no significant difference was found between the acyclovir and model groups (Figure 8A). On day 9, the expressions of gD, VP16, and ICP5 in the acyclovir group and JZ-1 groups both decreased, but the expression of HVEM and Nectin-2 only decreased in the JZ-1 group (Figures 8B,C). No significant change was found in the expression of Nectin-1 in all the groups. The changes in vaginal and vulvar tissues were the same.

#### Effect of JZ-1 on the TLR signaling pathway in the vaginal and vulvar tissues of GH mice

**FIGURE 9:**
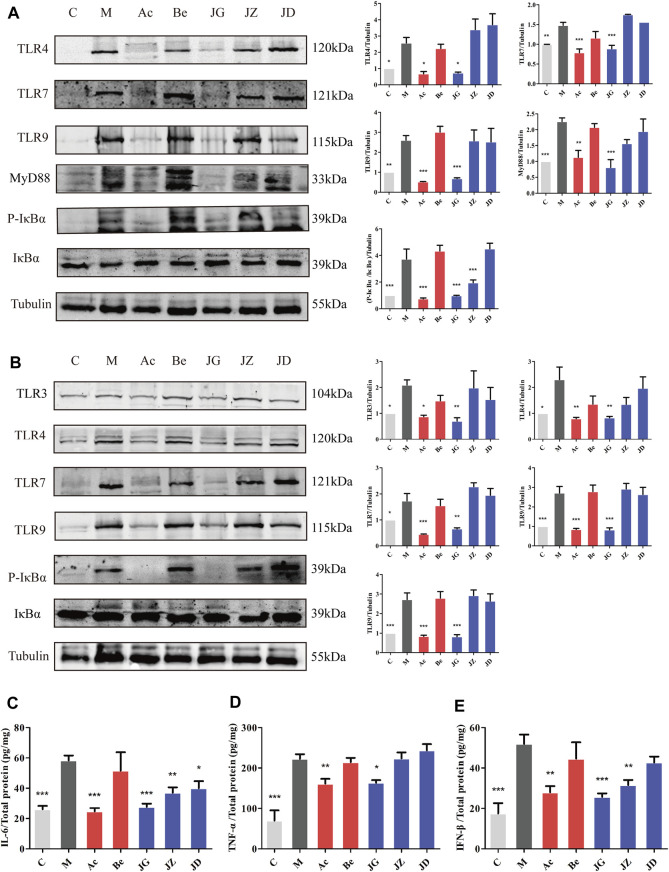
Effect of JZ-1 on the TLR signaling pathway in vaginal **(A)** and vulvar **(B)** tissues of GH mice. On day 9 after modeling, the key proteins of the TLR signaling pathway, including TLR3, TLR4, TLR7, TLR9, MyD88, P-IκBα, and IκBα were measured by western blot. The inflammatory cytokine contents in vaginal tissue, including IL-6 **(C)**, TNF-α **(D)**, and IFN-β **(E)**, were determined by ELISA. JZ-1 downregulated the activation of the TLR signaling pathway and the expression of inflammatory cytokines. C, control group; M, model group; Ac, acyclovir group; Be, berberine group; JG, JZ, JD: 2.5, 1.5, and 0.5 g/ml JZ-1 group. *p<0.05, **p<0.01, ***p<0.001 *vs.* M group.

TLR is the molecular basis of pathogen recognition. The adsorption and penetration of HSV-2 can activate the TLR signal pathway in vaginal and vulvar tissues (Martinez-Martin and Viejo-Borbolla, 2010). On day 9, the expression of TLR3, TLR4, TLR7, TLR9, and P-IκBα/IκBα in the vaginal tissue (Figure 9A), TLR4, TLR7, TLR9, MyD88, and P-IκBα/IκBα in vulvar tissue (Figure 9B) and IL-6, IFN-β, and TNF-α in vaginal tissue (Figures 9C–E) were increased in the model group and decreased in the acyclovir group and JZ-1 groups, particularly in the 2.5 g/ml JZ-1 group.

### Effect of JZ-1 on the Immune Response of GH Mice

**FIGURE 10:**
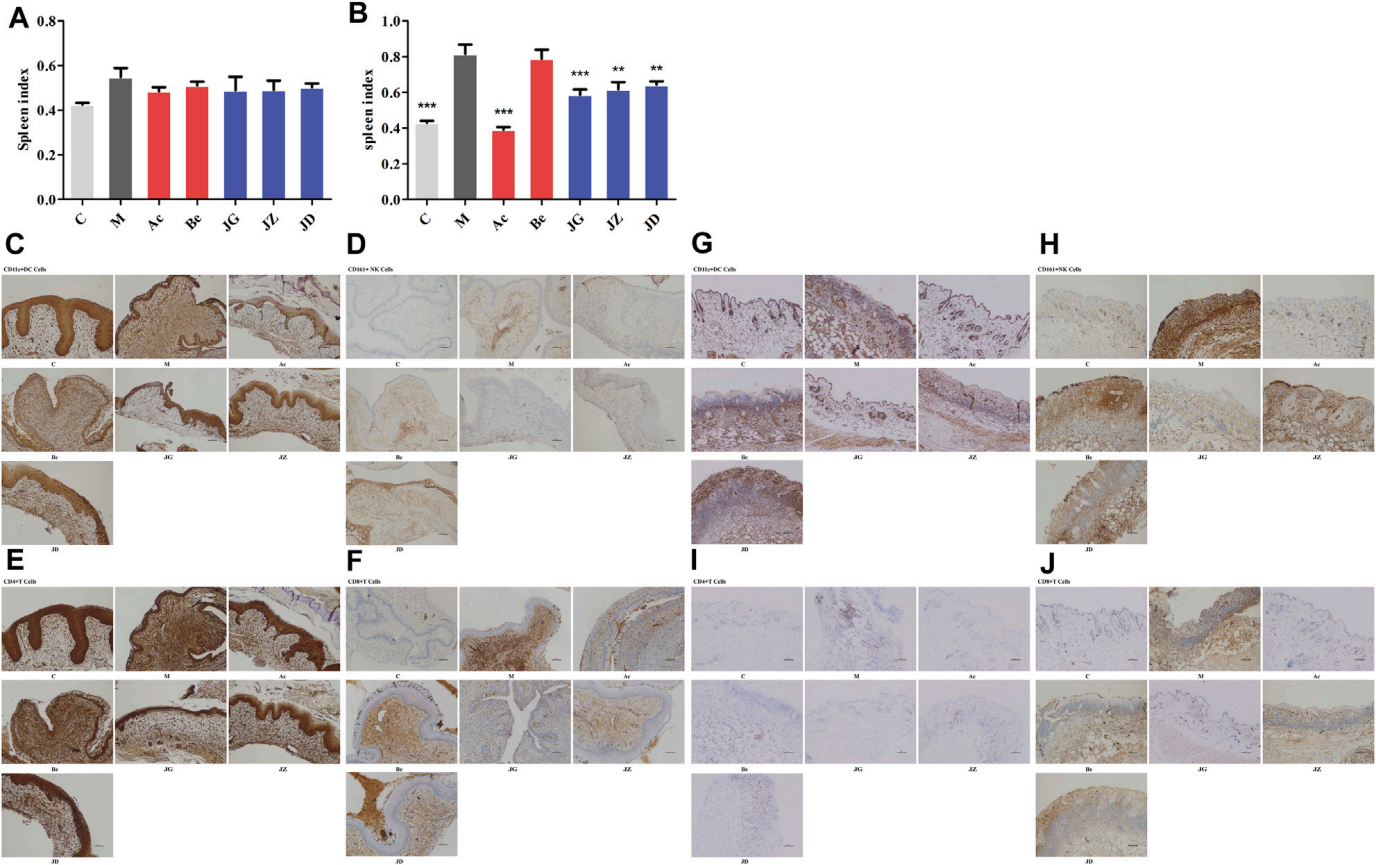
Effect of JZ-1 on the immune response of GH mice. On 12 h **(A)** and day 9 **(B)** after modeling, the spleen index of GH mice was measured. Moreover, the immune cell infiltration in vaginal **(C–F)** and vulvar **(G–J)** tissues of GH mice on day 9 were detected by immunohistochemistry staining. JZ-1 reduced the spleen index and infiltration of immune cells. C, control group; M, model group; Ac, acyclovir group; Be, berberine group; JG, JZ, JD: 2.5, 1.5, and 0.5 g/ml JZ-1 group. *p<0.05, **p<0.01, ***p<0.001 *vs.* M group.

The activation of the TLR signal pathway and the production of cytokines can further affect the immune cell infiltration and systemic immune response of the host (Uyangaa et al., 2014). Spleen index is one of the important indexes of immune function to some extent (Li et al., 2018). At 12 h, no significant difference was found in the spleen index among these groups (Figure 10A). On day 9, the spleen index (Figure 10B) and infiltration of immune cells (Figures 10C–J) in the vaginal and vulvar tissues of mice were both increased in the model group and decreased in the acyclovir and JZ-1 groups, particularly in the 2.5 g/ml JZ-1 group.

No significant difference was shown between the berberine and model groups in all the above results.

## Discussion

The Chinese herbal prescription Jieze-1 is used clinically for female lower reproductive tract infections diseases. *In vitro* experiments show that JZ-1 exhibits a highly effective anti-genital herpes effect (Duan et al., 2020). JZ-1 also showed a significant anti-HSV-2 effect in a mouse model of genital herpes in this study. In order to understand the anti-HSV-2 effect and mechanism of JZ-1 *in vivo*, we firstly established and optimized the GH mouse model with multiple batches of mice and recorded the changes in the target indexes over time within 2 weeks after modeling to evaluate the severity of the disease and drug efficacy accurately. HSV-2 infection is a sequential process started with the adsorption and penetration of HSV-2 into host cells (Luo et al., 2015). Then HSV-2 is recognized by Toll-like receptors and produces cytokines that regulate the systemic immune response, including immune cell infiltration (Uyangaa et al., 2014). So we chose to explore the expression of membrane fusion-related proteins, TLR signaling pathway-related proteins, cytokines, and immune cells in GH mice to study the mechanism of the anti-HSV-2 effect of JZ-1.

Firstly, the results gave us a good understanding of the pathogenesis of GH in mice. According to the results, this process can be divided into three stages. The first stage is day 0 to day 1. The modeling operation made the vaginal mucosa thin and caused acute inflammation. The expression of inflammatory factors in the vagina of mice increased. The second stage is day 1 to day 5. The acute inflammation of the vaginal tissue caused by the modeling operation will decrease slightly, resulting in a slight reduction in inflammatory factors and a slight increase in weight on day 5, which is still different from the normal state. Viral infections in vaginal tissues continue to rise. The third stage is from day 5 to day 14. Day 5 is a turning point. On the one hand, the mouse vaginal virus load reached a peak from day 3 to day 5. The high-load virus began to destroy healthy vaginal tissue, causing visible pathological manifestations and a large amount of cytokine secretion and immune cell infiltration. The inflammation and immune response increased sharply and rapidly from day 5 and reached a peak on day 9. On the other hand, vulvar ulcers gradually developed. After adsorption and penetration, the virus migrated to the nerve sheath and resided in sensory neurons for 1–3 days (Koelle and Wald, 2000). After 4–5 days, the virus infected the surrounding neurons and returned to the out peripheral epidermis, leading to clinical vulvar primary infection syndrome (Koelle and Wald, 2000). All symptoms reached a peak on day 9. The vaginal viral load (HSV-2 gB mRNA) showed a downward trend from day 5 that may be related to the gradually activated immune response (Dai et al., 2018). The viral load reached a low point from day 12 to day 14, and the vulvar ulcer began to heal. The body weight changed with symptoms.

HSV-2 infection is started with the adsorption and penetration of HSV-2 into host cells (Luo et al., 2015). Multiple glycoproteins, such as gB, gD, gH, gL, and gJ, play essential roles in viral entry, as well as immune-modulation and immune-escape (Roller and Roizman, 1992; Retamal-Díaz et al., 2015). Studies *in vitro* have demonstrated that HSV-2 can fully adhere and penetrate host cells within 5 min of incubation with vaginal epithelial cells (Duan et al., 2020). One to 2 h after infection, the viral capsid migrates to the nucleus mediated by microtubules, dynamic proteins and releases chromatin into the nucleus (Ibáñez et al., 2018). Immediate early proteins such as ICP0, ICP4, and ICP22 were mostly produced at 4–8 h after infection (Ibáñez et al., 2018). Early proteins such as TK, ICP8, and Uracil were mostly produced at 8–12 h after infection (Ibáñez et al., 2018). Late proteins such as gD, VP16, and ICP5 were mostly produced at 12–18 h after infection (Ibáñez et al., 2018). At 20–23 h after infection, the viral structural proteins form the nucleocapsids, wrap the virus DNA to get out of the nucleus, which was further processed in the cytoplasm to form offspring virons and released to extracellular (Ibáñez et al., 2018). Thus, we selected 12 h after modeling as the early stage of GH in mice. At this time, many virions adsorbed into and penetrated vaginal cells, and the expression of membrane fusion-related proteins in vaginal tissue increased significantly. Therefore, we chose 12 h after modeling as the time point to explore the anti-HSV-2 membrane fusion effect of JZ-1.

Immune and non-immune cells express a lot of molecular sensors that detect virus components which can promote the rapid antiviral responses that inhibit viral replication and propagation (Mogensen, 2009). Once the virus comes into contact with the host cells, host receptors such as TLRs will sense these stimuli and activate the Toll-like receptor signaling pathway, resulting in the expression of inflammatory cytokines with antiviral activity (Uyangaa et al., 2014). Toll-like receptors play an important role in both HSV-2 infection *in vivo* and *in vitro*. Human TLR3-deficient CNS cells have impaired intrinsic immunity to HSV-1 (Lafaille et al., 2012). TLR2 and TLR9 synergistically control herpes simplex virus infection in the brain (Sorensen et al.). TLR4-MyD88/Mal-NF-kB axis is involved in infection of HSV-2 in human cervical epithelial cells (Liu et al., 2013). Activation of plasmacytoid dendritic cells (PDC) is caused by the involvement of nucleotide receptor-like receptors (TLRs) 7 and 9, which play a key role in antiviral immunity (Gotoh et al., 2010). Our experiments found that essential proteins of the TLRs signaling pathway were indeed involved in HSV-2 infection. We also found time-related features of the expression of each protein. TLR4 was located on the cell surface and was the first to be expressed after modeling. TLR3, TLR7, and TLR9 were located intracellular, and their expression were presented in a time-dependent manner. The phosphorylation of IκBα indicates the release of NF-κB inhibition (Hayden and Ghosh, 2004). NF-κB enters the nucleus and promotes the synthesis of cytokines such as IL-6, TNF-α, and IFN-β, which can regulate the immune response (Dunne and O’Neill, 2003; Hayden and Ghosh, 2004; Yamamoto et al., 2004). IL-6 can promote the growth and differentiation of original bone marrow-derived cells and plays a vital role in a series of processes such as the maturation, activation, proliferation, and immune regulation of immune cells (Tanaka et al., 2014). The primary role of TNF-α is to regulate the function of immune cells. It can induce chemokines and adhesion molecules, activate endothelial cells and promote T cells to produce various inflammatory factors which promote the occurrence of inflammation. In addition, natural killer (NK) can also receive signals through TNFR to amplify division, survival, and cytokine production (Aggarwal et al., 2012). IFN-β can inhibit viral growth by interfering with viral DNA replication, significantly enhance the killing activity of NK cells, and regulate T cells (Haji Abdolvahab et al., 2016). Immune cells regulated by these cytokines can further exert immune responses. NK cells are one of the innate immune cells that are capable of sensing and destroying HSV-2-infected cells (Céspedes et al., 2013). DC antigen presentation to T cells can lead to a process that involves DC-T cell interactions, resulting in the activation of T cells (Retamal-Díaz A. R. et al., 2017). T cells can regulate immune and non-immune cells, eventually killing infected cells (González et al., 2007; Tognarelli et al., 2019). Besides, NK cells, T cells, and DCs can all produce interferon to inhibit virus replication (Elboim et al., 2013). In our study, the TLRs and the infiltration of immune cells in the vagina increased significantly as the symptom score of mice reached a peak on day 9. Considering the above results, we also chose the 9th day after modeling as the time point to explore the anti-GH effect and mechanism of JZ-1 *in vivo*.

Based on the above meticulous explorations, we researched the effect of JZ-1 on HSV-2 infection and explored the underlying mechanism by examining different targets*.* First, weight changes and vulvar symptoms are the most intuitive indicators to evaluate the efficacy of antiviral drugs. JZ-1 and acyclovir effectively alleviate the symptoms and weight loss of GH mice, indicating JZ-1 has the same anti-HSV-2 effect as acyclovir *in vivo* experiments. The role of JZ-1 is not just improving symptoms. We measured the viral load in the vagina, vulva, and spinal cord, revealing that JZ-1 effectively inhibited the viral load. A positive correlation exists between the spinal cord viral load and virus recurrence probability (Kollias et al., 2015). No viral load was detected in the spinal cord of GH mice 12 h after infection because the genome of HSV reaches the spinal cord 24 h after infection (Steiner et al., 1990). JZ-1 can significantly reduce the viral load of the spinal cord on day 9; whether JZ-1 can play a role in reducing the recurrence of GH or alleviating the symptoms of recurrence deserves further study. On the other hand, HSV-2 infection transmits through the intermittent release of reproductive tract shedding, with significant potential risks. The amount of vaginal viral shedding is vital in evaluating GH’s infectivity (Sacks et al., 2004). The results showed that JZ-1 significantly reduces the amount of vaginal viral shedding in GH mice, suggesting the possibility of its effective reduction of cross-infection risk.

In order to confirm the anti-HSV-2 effect of JZ-1 *in vivo* from the histological level, the histopathological changes of vagina and vulva of mice were observed under hematoxylin-eosin staining and electron microscope, respectively. The results of hematoxylin-eosin staining suggested that JZ-1 could significantly alleviate the vacuolar degeneration and necrosis of vaginal and vulva tissues caused by the virus, reduce the infiltration of inflammatory cells, and promote tissue keratinization and repair. The typical morphology of organelles is essential for cells to perform normal physiological functions. Ultrastructure of cells of the genital herpes mouse model treated with JZ-1 revealed the regular appearance of organelles, suggesting the cells were protected against HSV-2 infection, a phenomenon that needs to be further explored.

In order to understand the anti-adsorption and anti-penetration effect of JZ-1, we detected the expression of membrane fusion-related proteins in the vaginal and vulva tissues of mice. By significantly downregulating the expression of membrane fusion-related proteins, JZ-1 reduces the adsorption and penetration of HSV-2. In cells with the Nectin-2 receptor, the membrane fusion of HSV-2 is mainly mediated by Nectin-2 instead of Nectin-1 (Fujimoto et al., 2017). Thus, the expression of Nectin-1 showed no difference among all the groups. Acyclovir did not change the Nectin-2 expression because it only acts on the replication stage (Field et al., 2013). The downregulation of VP16 expression, a transactivator that can promote viral gene transcription (Thompson and Sawtell, 2019), suggests that JZ-1 may also inhibit HSV-2 genome replication *in vivo*. As a member of the tumor necrosis factor receptor family, HVEM is not only expressed in vaginal epithelial cells but also in inactivated immune cells (Hu et al., 2017). The downregulation of HVEM suggests that JZ-1 not only inhibits membrane fusion but also regulates immune response.

Next, based on the Toll-like receptor signaling pathway and immune cells, we conducted our research on the immune responses. The HSV-2 of the model group fully activated the TLR signaling pathway. We first explored the expression of Toll-like receptors in the vaginal area where the virus was inoculated. Then, for the ulcer lesions of the vulva, we specifically explored the expression of TLR-MyD88 signaling pathway-related proteins. JZ-1 downregulates the pattern recognition of viruses by TLRs, inhibits the expression of the TIR domain adapter MyD88 in vulvar lesions, and reduces the phosphorylation of IκBα to inhibit NF-κB transduction into the nucleus, significantly reducing the release of cytokines such as IL-6, IFN-β, and TNF-α, and inhibiting the inflammatory response caused by the excessive immune response. So does JZ-1 further suppress the excessive immune response? We observed it by detecting the spleen index and immune cell infiltration.

In the immune system, the spleen is the largest peripheral immune organ, and the morphology of the spleen can directly affect the immune function of the body (Gu et al., 2014). At 12 h, only local inflammation caused by physical friction occurred in mice, so the spleen index among all the groups showed no difference. On day 9, the mice reached a peak of symptoms with immune dysfunction. Their body weight decreased, and the spleen enlarged compensatively, causing an increased spleen index and massive infiltration of immune cells in vaginal and vulvar tissues. JZ-1 can indeed reduce the spleen index and infiltration of NK, DC, and T cells.

JZ-1 is a traditional Chinese herbal prescription with many components. We have identified nine of them by HPLC. They were D-(-)-quinic acid, citric acid, trigonelline, caffeic acid, apocynin, taxifolin, ferulic acid, luteolin, and berberine. According to some reports, trigonelline (Ozçelik et al., 2011), apocynin (Hu et al., 2011), caffeic acid (Ikeda et al., 2011; Ozçelik et al., 2011; Utsunomiya et al., 2014), luteolin (Ojha et al., 2015; Rittà et al., 2020) and berberine (Chin et al., 2010) have anti-HSV effects. And quinic acid can resist hepatitis B virus (Wang et al., 2009) and dengue virus (Zanello et al., 2015). A disinfectant containing 0.2% citric acid can fight the foot-and-mouth disease virus (Hong et al., 2015). Taxifolin can fight hepatitis C virus (Pisonero-Vaquero et al., 2014). On the one hand, apocynin (Nam et al., 2016), trigonelline (Khalili et al., 2018), luteolin (Chen et al., 2018) inhibited Toll-like receptor-4-mediated activation of NF-κB. Ferulic acid protected against porcine parvovirus infection-induced apoptosis by suppressing the NF-κB and TLR4 (Ma et al., 2020). Berberine inhibits the expression of TNF-α, IL-6, TLR 4, and TLR 9 in the early phase of sepsis in rats (Li et al., 2015). Berberine also suppressed the viral infection-induced up-regulation of the TLR7 signaling pathway, such as TLR7, MyD88, and NF-κB (Yan et al., 2018). On the other hand, caffeic acid and ferulic acid showed the ability to enhance the killing activity of T cells and NK cells (Kilani-Jaziri et al., 2017). Citric acid plays an important role in activating dendritic cells (Ryan and O’Neill, 2017). Apocynin can inhibit cytokine production of CD8^+^ T cells (Nam et al., 2014). Luteolin reduces the frequency of mature dendritic cells and CD4+/CD8+ T cells (Ye et al., 2019). Berberine can significantly reduce T lymphocyte and NK cell infiltration (Li et al., 2020). Meanwhile, berberine can also inhibit the maturity of dendritic cells and reduce the secretion of IL-6, IL-1β, and IL-23 by dendritic cells (Yang et al., 2013). Taken together, these chemical components of JZ-1 may function synergistically to HSV-2, Toll-like receptor pathway, and immune cells. These reports support our results.

Monarch medicine plays a significant role in Chinese herbal prescription (Xie, 2016). *Phellodendron chinense* C.K.Schneid. is the monarch medicine of JZ-1. Berberine, the identified component of JZ-1 monarch medicine (Chinese Pharmacopoeia Commission, 2020), is the primary indicator of quality control in the preparation process of JZ-1 (Ma et al., 2011). There are many reports on the antiviral and anti-inflammatory effects of berberine (Warowicka et al., 2020). It was used in our study to determine whether it can exert the same antiviral effect as the Chinese herbal prescription JieZe-1. However, 891.8 μM (0.3 mg/ml) of berberine gel showed no antiviral effect *in vivo*, suggesting that the components in the Traditional Chinese Medicine prescription are not necessarily effective. Whether it is related to the concentration or administration and whether the other components in the JZ-1 have antiviral effects are worthy of further verification. This result also suggests that the antiviral effect of the Chinese herbal prescription JieZe-1 may be exerted by the interaction of various components, reflecting the scientific implication of TCM. This is also the charm of the pharmacology of traditional Chinese herbal prescriptions.

## Conclusion

In summary, the treatment of GH mice with JZ-1 gel improved the symptoms that have been confirmed by histopathological and cell ultrastructural analysis. JZ-1 can also reduce the vaginal, vulvar, and spinal cord viral load and vaginal virus shedding. The potential mechanism is associated with the inhibition of membrane fusion, the TLR signaling pathway, inflammatory cytokines, and cellular immunity. The above studies provide experimental evidence to reveal the effect and partial molecular mechanism of the Chinese herbal prescription JieZe-1 on anti-HSV-2 in GH mice. The anti-HSV-2 efficacy of JZ-1 is slightly less than that of acyclovir. However, it also shows preventive and therapeutic effects on multiple lower reproductive tract infectious diseases. The excellent application prospect of JZ-1 is worthy of further research and exploration.

## Data Availability

The raw data supporting the conclusions of this article will be made available by the authors, without undue reservation.

## Abbreviations

DCs, dendritic cells; gD, glycoprotein D; gB, glycoprotein B; gH, glycoprotein H; gL, glycoprotein L; GH, genital herpes; HPLC, High-performance liquid chromatography; HSV-2, herpes simplex virus type 2; HVEM, herpesvirus entry mediator ligand protein; ICP5, earliest virus-specific infected cell polypeptides 5; IFN-β, interferon-β; IL-6, interleukin-6; IκBα, nuclear factor-kappa-B inhibitor alpha; JZ-1, JieZe-1; MyD88, myeloid differentiation factor 88; NK cell, natural killer cell.; TLR, toll-like receptor; TNF-α, tumor necrosis factor-α; VP16, viral protein 16.
