# Supplementary material for: The Chinese Herbal Prescription JieZe-1 Inhibits Membrane Fusion and the Toll-like Receptor Signaling Pathway in a Genital Herpes Mouse Model
Source: Front Pharmacol. 2021 Sep 24;12:707695. doi: 10.3389/fphar.2021.707695 (PMC8497740; doi:10.3389/fphar.2021.707695)
Supplement: Supplementary file 1 [file DataSheet1.PDF]

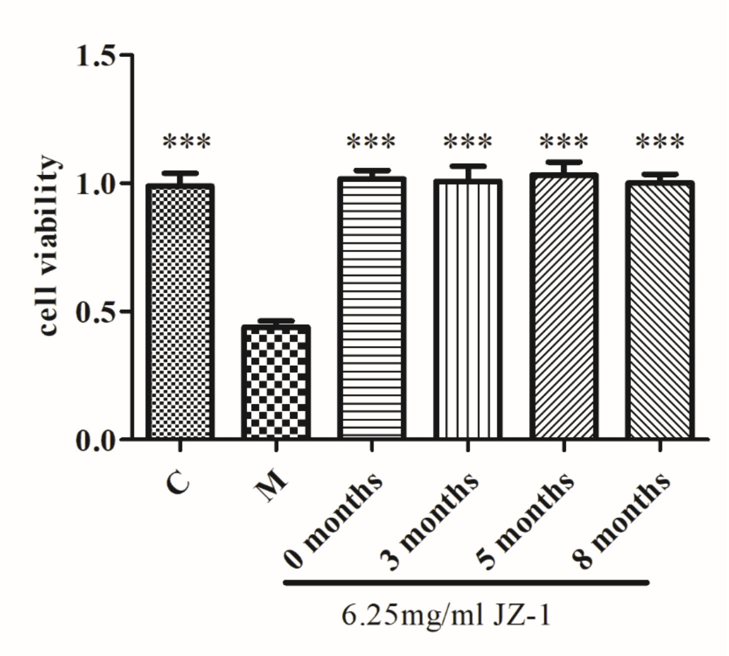

Supplementary Figure 1: Same batch of JZ-1 showed stable anti-HSV-2 effect at different time within 8 months

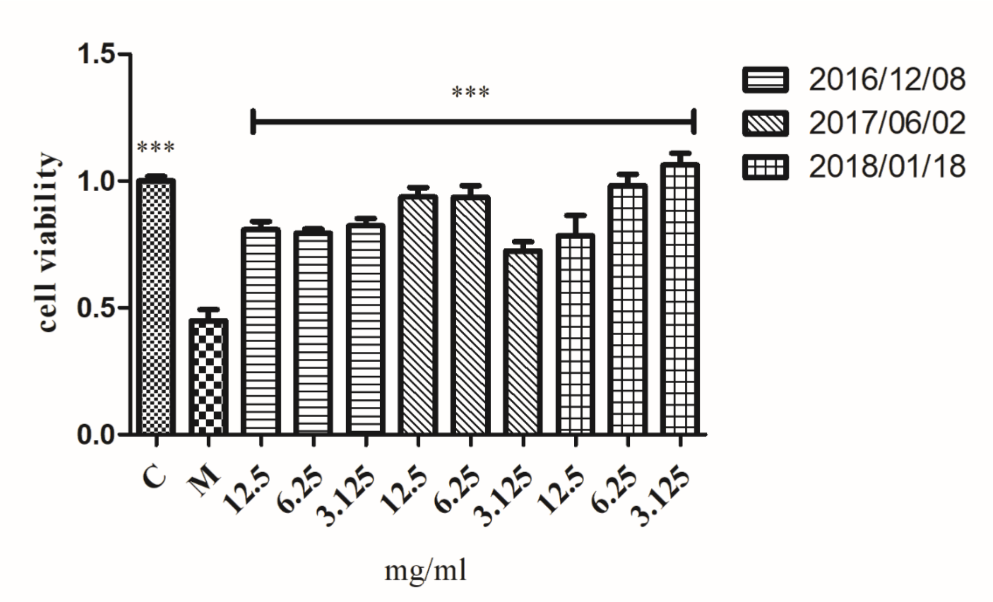

Supplementary Figure 2: Different batches of JZ-1 showed stable anti-HSV-2 efficacy

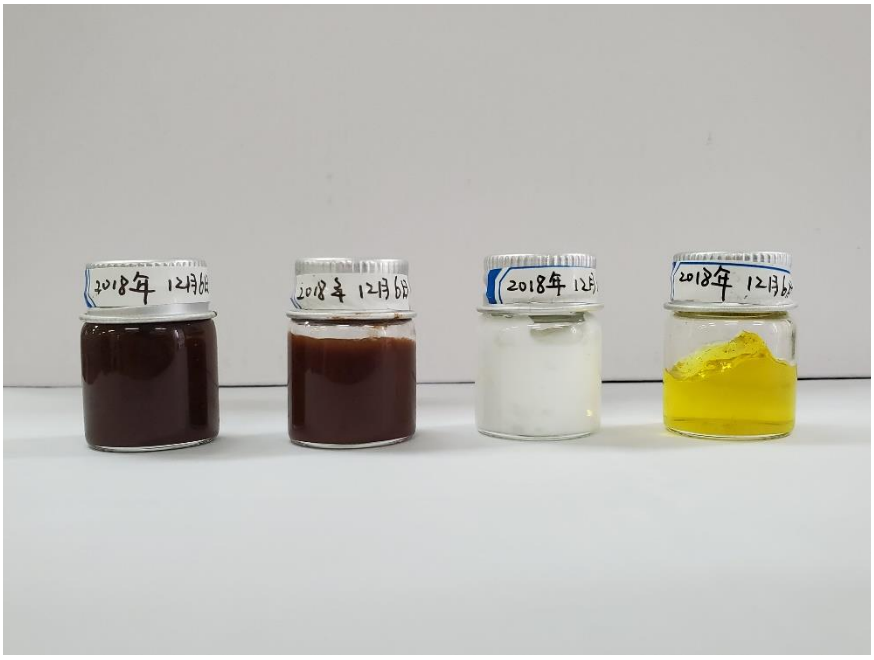

Supplementary Figure 3: The vaginal gels made in December 2018 exhibit a stable nature
